# Supplementary material for: High-Quality Genome Assembly and Genome-Wide Association Study of Male Sterility Provide Resources for Flax Improvement
Source: Plants (Basel). 2023 Jul 26;12(15):2773. doi: 10.3390/plants12152773 (PMC10421198; doi:10.3390/plants12152773)
Supplement: Supplementary file 1 [file plants-12-02773-s001.zip › Supplementary Tables.pdf]

Table S1. Survey statistic results of flax.

Species	Total base (Gb)	K-mer	K-mer number	K-mer depth	Genome size (Mb)	Heterozygous ratio (%)	Repeat ratio (%)
flax	49.82	111	13,285,160,936	9.78	506.4	0.02	35.4

Table. S2 Summary of the final assembly of the flax genome

PacBio+Hi-C				
Title	Contig Length (bp)	Scaffold Length (bp)	Contig Number	Scaffold Number
Total	473553829	474080129	6099	836
Average	77644	567081	-	-
Max	7684812	42825733	-	-
N50	911211	31716147	110	7
N60	431614	31109343	188	9
N70	163143	30010956	368	10
N80	54833	26736738	908	12
N90	21783	23704220	2333	14

Table S3. Length of chromosomes

Chromosome ID	Length (bp)
Chromosome 1	23704220
Chromosome 2	26736738
Chromosome 3	27112812
Chromosome 4	31109343
Chromosome 5	31716147
Chromosome 6	32082761
Chromosome 7	30010956
Chromosome 8	31576709
Chromosome 9	35763222
Chromosome 10	42825733
Chromosome 11	36221436
Chromosome 12	34743211
Chromosome 13	32993160
Chromosome 14	19914620
Chromosome 15	26490608
Average	30866778
Total	463001676 (97.66%)

Table S4. Coverage statistics of flax genome

		Percentage
Reads	Mapping rate (%)	99.70%
Genome	Coverage (%)	99.74%
	Coverage at least 4X (%)	99.59%
	Coverage at least 10X (%)	99.49%
	Coverage at least 20X (%)	99.38%
	Average sequencing depth	184.87X

Average sequence depth: The average depth of each base on the genome that is covered by reads;

Coverage：The proportion of genomes that were covered by reads;

Table S5. Assessment the gene coverage rate using BUSCO.

Species	BUSCO notation assessment results
flax	C:95.9%[S:28.3%,D:67.6%],F:1.1%,M:3.0%,n:2121

BUSCO notation: C: Complete BUSCOs; S: Complete and single-Copy BUSCOs; D: Complete and duplicated BUSCOs; F: Fragmented BUSCOs; M: Missing BUSCOs; n: Total BUSCO groups searched.

Table S6. Summary statistics for the Neiya No. 9 genome assembly in comparison with the CDC Bethune v2 and YY5 v2.0 reference genomes

Genomic feature	Neiya No. 9	YY5 v2.0	CDC Bethune v2
Total Assembly size	474.08 Mb	454.96 Mb	316.17 Mb
Number of contigs	836	337	26,086
Largest contig	42.83 Mb	33.98 Mb	151.81 Kb
Contig N50 length	0.91 Mb	9.61 Mb	22.12 Kb
Length of contigs anchored to chromosomes	97.66%	93.00%	100.00%
Number of gaps	5263	76	26,071
Length of gaps	11.60 Mb	7,523 bp	46.58 Mb
GC content	38.09%	38.89%	39.51%
Complete BUSCOs (genome)[a]	95.90%	94.40%	93.50%
LTR assembly index (LAI)	15.83	14.29	9.39
Intact LTR-RTs	1435	1444	293
Repetitive sequences	187.16 Mb / 39.5%	251.86 Mb / 55.36%	92.37 Mb / 29.21%
Protein-coding genes/transcripts	32,786 / 32,786	49,616 / 52,207	43,471 / 43,484

[a]The analysis from comparisons with the eudicotyledons_odb10 database

Table S7. Assessment the assembly continuity using LAI.

Assembly	Intact	Total	raw_LAI	LAI
This Study	0.0167	0.1282	13.06	15.83
CDC Bethune	0.0043	0.0796	5.42	9.39

Table S8. Summary of TE contents in flax genome.

Type	Length (bp)	Percent (%)
DNA	63,518,703	13.41
LINE	11,495,286	2.43
SINE	331,541	0.07
LTR	101,239,032	21.38
Low complexity	2,132,228	0.45
Simple repeat	4,864,312	1.03
Satellite	3,583,304	0.76
Unspecified	29,381,123	6.20
Total	187,164,406	39.52

Table S9. The statistical results of non-coding RNA of flax genome.

Type		Copy	Average length (bp)	Total length (bp)	% of genome
tRNA		1,952	73.00	144014	0.0304
catalytic intron		513	99.89	51246	0.0108
small nucleolar RNA		518	106.6	55217	0.0117
spliceosomal RNA		224	141.51	31699	0.0067
microRNA		168	122.88	20643	0.0044
rRNA	18S	216	1802.90	389,427	0.0822
	28S	211	3624.56	764,782	0.1615
	5.8S	240	153.18	36,764	0.0078
	5S	6,311	111.53	703,850	0.1486

Table S10. The statistical results of gene function annotation of flax genome.

Database	Annotated Number	Annotated Percent (%)
NR	32,169	98.12
Swiss-Prot	27,777	84.72
KEGG	18,107	55.23
Pfam	29,546	90.12
GO	18,708	57.06
Annotated	32,613	99.47%
Total	32,786	-

Table S11. Genes used for gene family clustering in each species

Symbol	ScientificName	Gene Number
ath	*Arabidopsis thaliana*	27,628
gmx	*Glycine max*	55,897
mesc	*Manihot esculenta*	33,044
pop	*Populus trichocarpa*	41,335
sbra	*Salix brachista*	30,209
jcu	*Jatropha curcas*	27,619
rcu	*Ricinus communis*	31,221
lus	*Linum usitatissimum*	32,786
mtr	*Medicago truncatula*	50,444

Table S12. Statistical analysis of segregation of sterile phenotype in offspring of infertile plants.

Year	Hybridization method	Number of combinations	Total plants	Sterile plants	Fertile plants	chi-squared value	*P* value
2015	Open pollination	-	4740	2338	2402	0.83	>0.05
2016	Sterile × Fertile (B_1F_1)	81	678	332	346	0.28	>0.05
	Sterile × Fertile (F_1)	15	86	44	42	0.02	>0.05
2017	Sterile × Fertile (B_2F_1)	78	6014	2932	3082	3.69	>0.05
	Open pollination	-	1743	883	860	0.29	>0.05
