# Supplementary figures and images for: High-Quality Genome Assembly and Genome-Wide Association Study of Male Sterility Provide Resources for Flax Improvement
Source: Plants (Basel). 2023 Jul 26;12(15):2773. doi: 10.3390/plants12152773 (PMC10421198; doi:10.3390/plants12152773)

## Slide 1
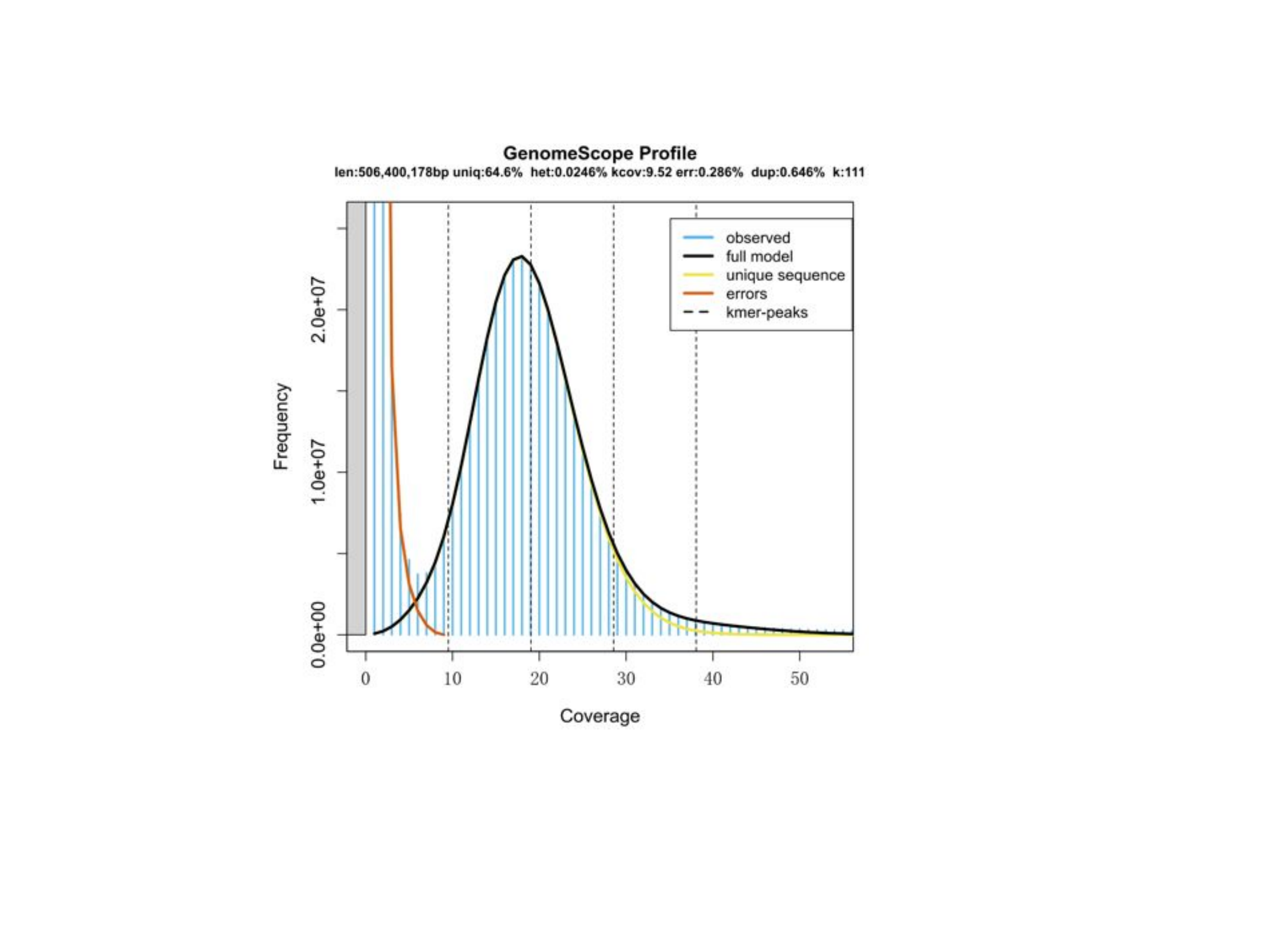

## Slide 2
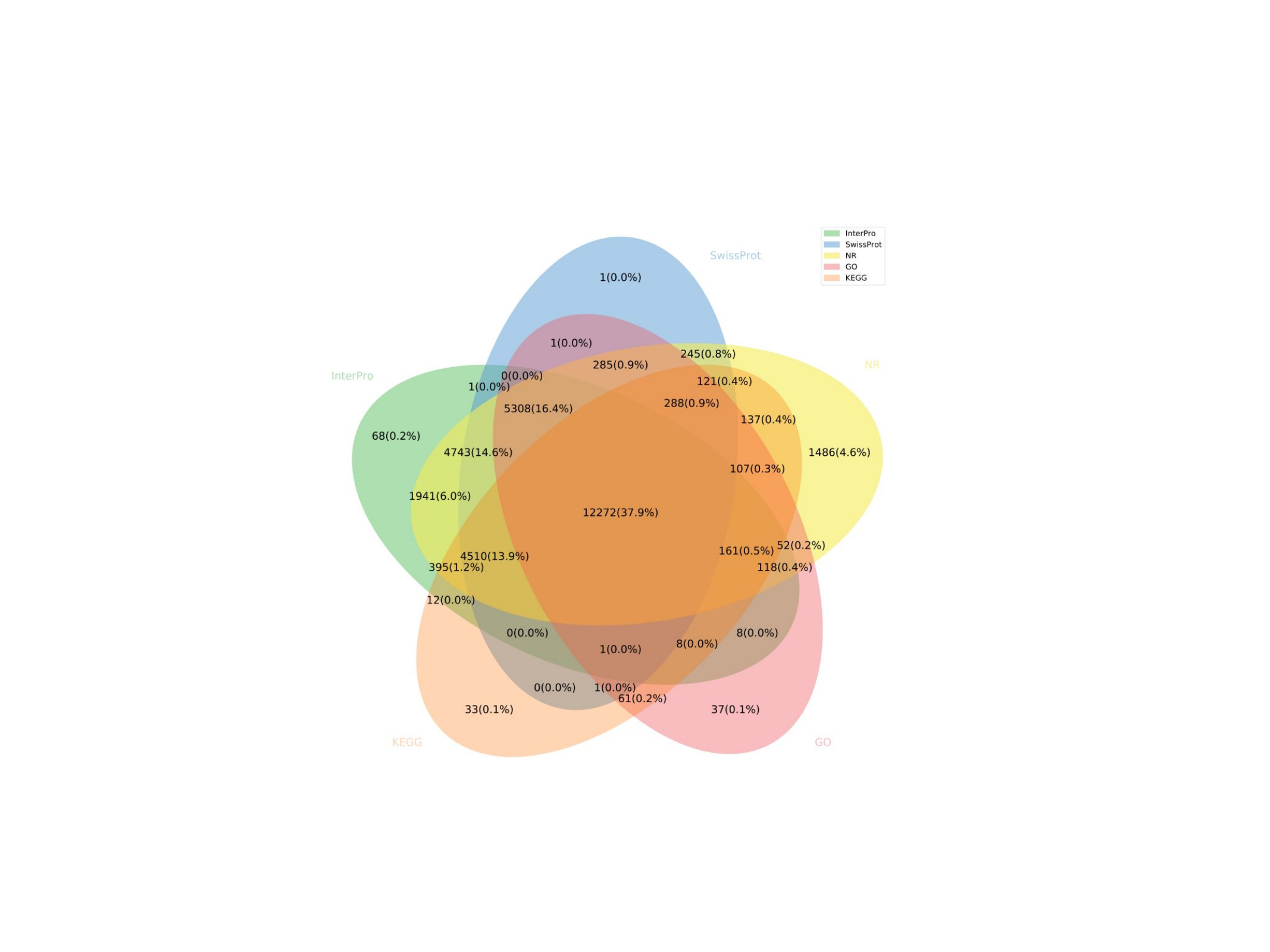

## Slide 3
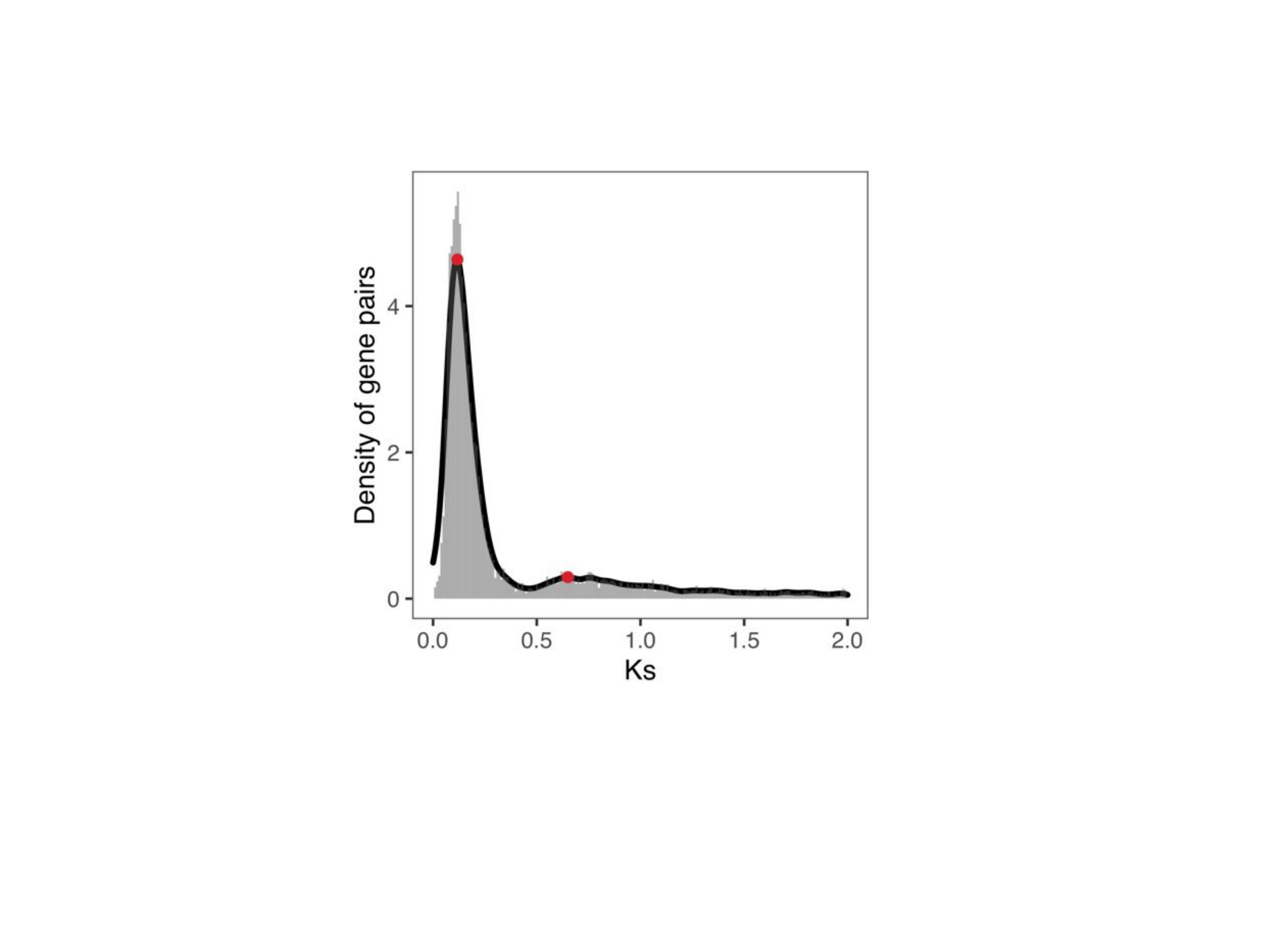

## Slide 4
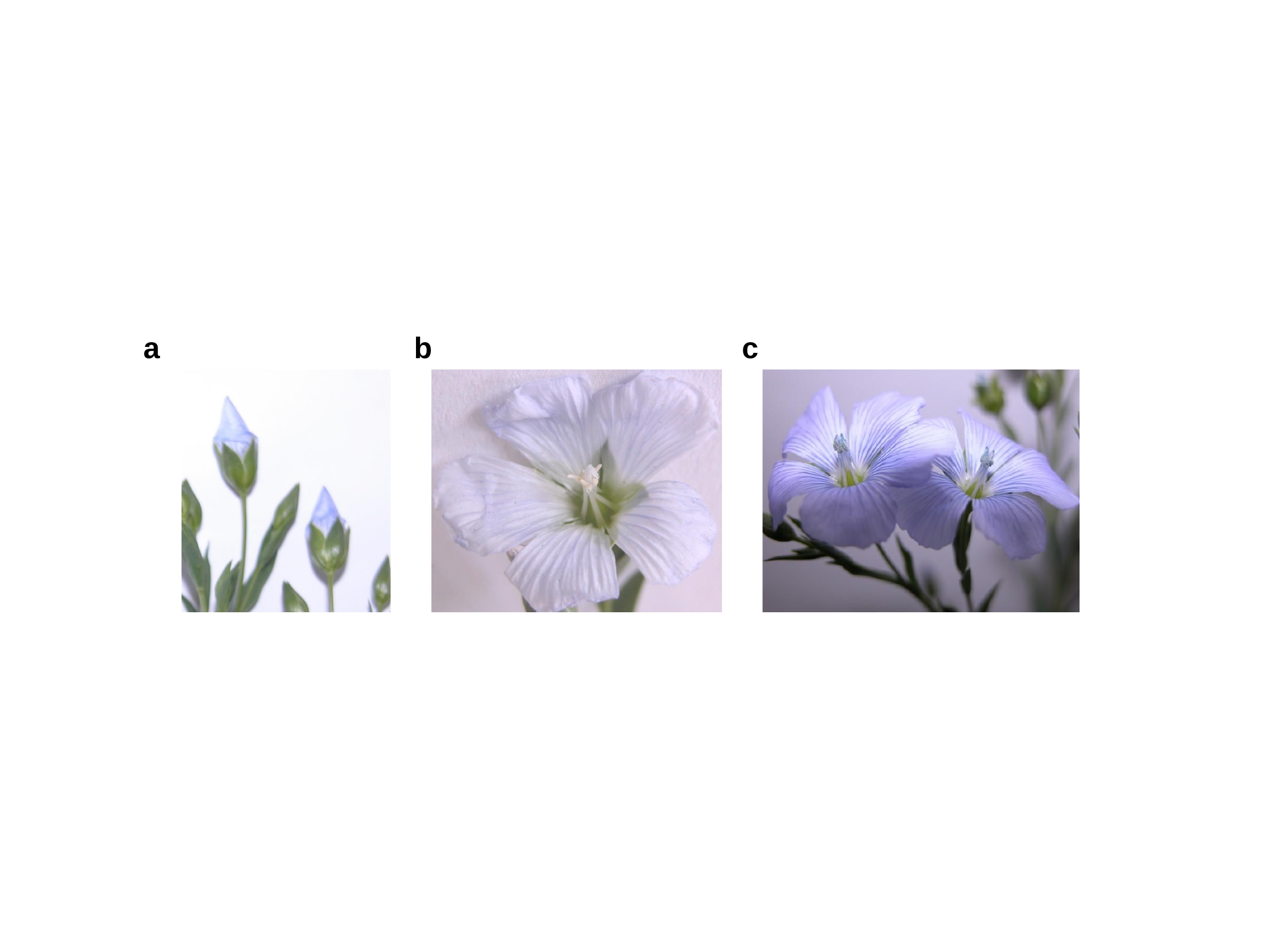

a
b
c

## Slide 5
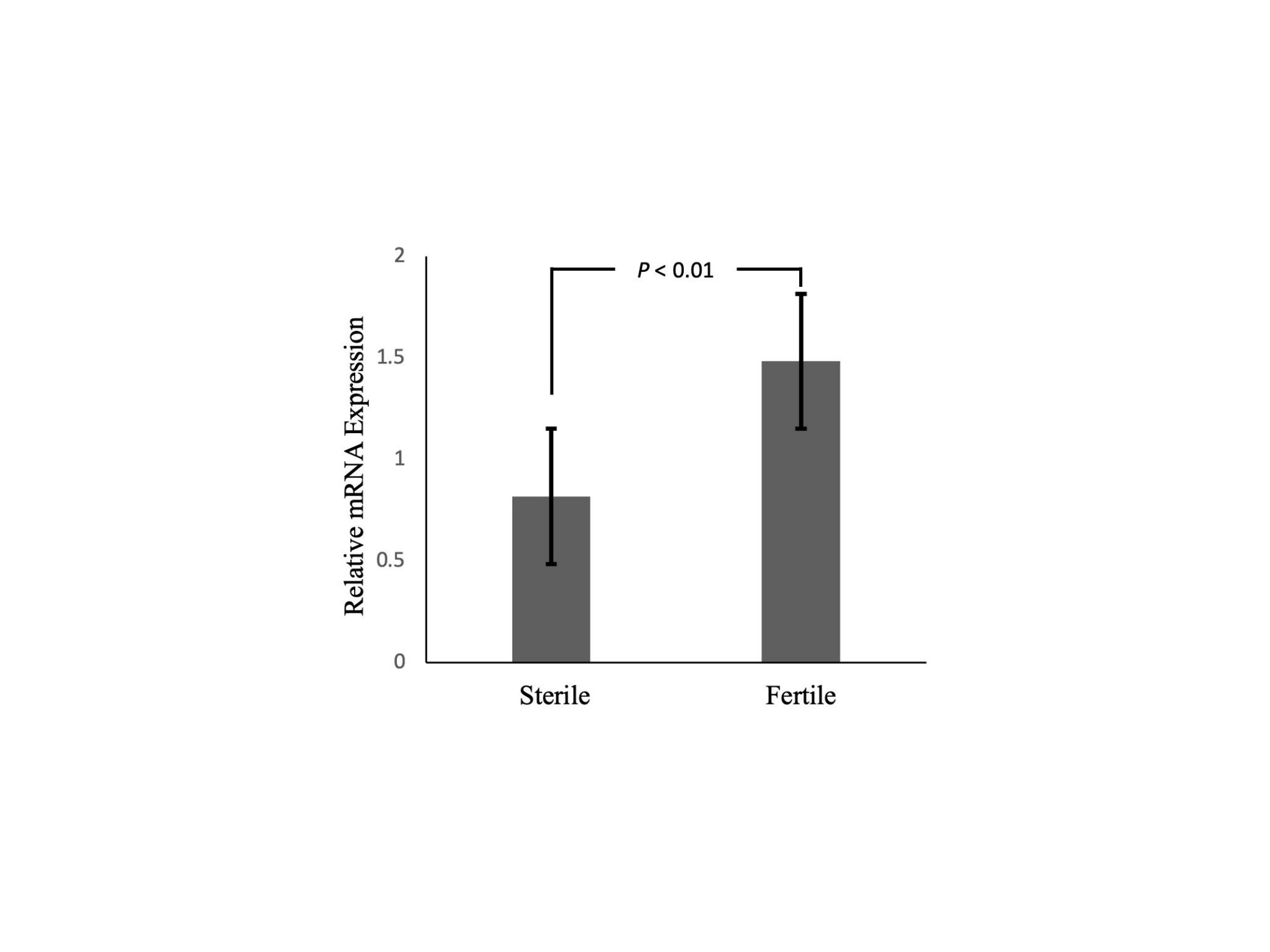

Supplement: Supplementary file 1 [file plants-12-02773-s001.zip › Supplementary Figures.pptx]
